# Cytogenetical and hematological analysis of chronic myelogenous leukemia patients with a novel case 52XX, t (1;9;22) (q23.3; q34; q11.2), +6, +8, i(9) (q10;q10), +18,+19,+21+der22 t(9;22)(q34;q11)

**DOI:** 10.1097/MD.0000000000031670

**Published:** 2022-11-11

**Authors:** Muhammad Asif, Abrar Hussain, Irfan Ali, Zarak Baloch, Mahmood Rasool, Niaz M. Achakzai

**Affiliations:** a Department of Biotechnology, BUITEMS, Quetta, Pakistan; b Office of Research Innovation and Commercialization, BUITEMS, Quetta, Pakistan; c Centre of Agricultural Biochemistry and Biotechnology, University of Agriculture Faisalabad, Faisalabad, Pakistan; d Faisalabad Medical University, Faisalabad, Pakistan; e Department of Medical Laboratory Technology, Faculty of Applied Medical Sciences, King Abdulaziz University, Jeddah, Saudi Arabia; f Department of Molecular Biology, DNA Section, Legal Medicine Directorate, Ministry of Public Health, Kabul, Afghanistan.

**Keywords:** additional chromosomal abnormalities, chronic myelogenous leukemia, complex/variants translocations, six additional chromosomes

## Abstract

(9;22) (q34; q11) translocation is appear in above ninety percent of chronic myelogenous leukemia patients while variant/complex translocations were observed in almost 5% to 8% chronic myelogenous leukemia (CML) positive cases. Gleevec (Imatinib Mesylate) is the first choice breakpoint cluster region (BCR)/ABL targeted oral therapy that produced a complete response almost in 71% to 80% of patients affected with CML. A complete blood count (CBC) of 37 patients was done during diagnosis, however only 21 showed abnormal CBC values which were selected for the study. Karyotyping study using bone marrow samples was performed on 21 CML patients for the conformation of 9;22, however, fluorescence in situ hybridisation was performed for the detection of the BCR–ABL fusion gene of 15 patients. Out of 21, 17 patients showed Ph-positive (9;22) (q34; q11) translocation. Sixteen CML patients showed standard translocation however only CML patients showed a three-way variant/complex translocation with six additional chromosomes, 52XX, t(1;9;22) (q23.3;q34;q11),+6,+8, i(9)(q10;q10), +18,+19,+21 + der22 t(9;22)(q34;q11)). Here we report we report a novel case of six additional chromosomes with the three-way translocation of 52XX, t(1;9;22) (q23.3;q34;q11),+6,+8, i(9)(q10;q10), +18,+19,+21 + der22 t(9;22)(q34;q11) in blast phase.

## 1. Introduction

Chronic myelogenous leukemia (CML) is a malignant hematopoietic stem cell disease, produced through the balance translocation between the long arms of chromosomes 9 and 22, so-called the Philadelphia chromosome.^[1]^ It is a result of a combination of two genes Breakpoint cluster Region (BCR) and Ableson (ABL) that form the fusion gene BCR–ABL that produces a chimeric protein, tyrosine kinase activity. This tyrosine kinase activity is accountable for regulating and keeping the adult tissues and helps in the signaling pathway and regulating cell division.^[2]^

The translocation 9 and 22 chromosome is established in up to 90% of CML-positive cases. On the other hand variant/complex translocations including one or more additional chromosomes instead of (9; 22)(q34; q11) were perceived only <8% of CML patients.^[1,3,4]^ However genetic additional abnormalities happen in <10% of chronic myeloid leukemia cases during the diagnosis stage and other 60% to 70% are detected in accelerated and blast crisis.^[5,6]^ In CML trisomy 19 has usually happened as a secondary abnormality, non-common as trisomy 8, (17q) and extra chromosome Ph chromosome. Trisomy 19 has been observed only in about 15% of CML cases.^[6–9]^

Imatinib Mesylate (Gleevec) is an orally designed BCR/ABL protein tyrosine kinase inhibitor against BCR/ABL, platelet-derived growth factor receptor (PDGF-R), and c-kit.^[10]^ This oral therapy is the first choice of treatment for almost 80% to 90% of CML patients of all phases.^[11]^ Imatinib Mesylate orally at 400 mg/daily dos indeciduate complete hematologic response in 95% of CML patients and complete cytogenetic responses in 61% to 90.^[12]^ After 18 months of follow-up, about 90% of CML patients are unable to transform to the accelerated or blastic phases. In conclusion, this drug has most effective for prolonged survival and better quality of life.^[13,14]^

Here we reported a novel case of ACAs with three way translocation in CML patient with 6 additional chromosome 52XX, t(1;9;22) (q23.3;q34;q11),+6,+8, i(9) (q10;q10), +18,+19, +21 + der22 t(9;22)(q34;q11).

## 2. Materials and methods

The current study was conducted between July 2014 and September 2020 in different Hospitals in Quetta and Allied Hospital Faisalabad. In this study, a total of 21 patients with CML were enrolled of which 11 were male and 10 were female. All participants in the current study completed a written informed consent form. The study was approved by BUITEMS’s local ethical committee in Quetta, Pakistan.

### 2.1. Hematological analysis

For complete blood cell count, we used a hematological analysis for the analysis of red blood cells, white blood cells (WBC), hemoglobin, mean corpuscular volume, platelet, and hematocrit.

### 2.2. Karyotyping analysis

Karyotyping analysis was executed by the GTG-banding method. For this purpose, bone marrow samples were studied in direct or short-term (24-hour) cultures, and 22 metaphases were examined. In addition, karyotypes were expressed by the International System for Human Cytogenetic Nomenclature.^[9]^

### 2.3. FISH analysis

The fluorescence in situ hybridisation test was executed by directly labeled dual color LSI/CEP probes provided by the supplier’s instructions (BCR/ABL– Oncor) for the recognition of BCR/ABL gene fusions. In this technique, 200 metaphase or interphase cells were counted.^[9]^

## 3. Results

Table 1 shows the general physical and CBC characteristics of patients affected by CML. The study was conducted on 21 patients of which 11 were male and 10 were female. The subjects were characterized into three age groups. Results reveal that a maximum of 10 (47.42%) CML patients were from the 31 to 45 age groups. Signs and symptoms like, anxiety 61.90%, depression 57.14%, swelling of the body 61.90%, spleen enlargement 47.60 %, and liver enlargement were detected in the CML patients. Table 1 also shows the results of WBC, hemoglobin, and platelets (PLTs). WBC normal values were seen in 2 (9.5%) patients; but 19 (90.47%) patients displayed a higher value of WBC, however, 13 (61.90%) patients showed lowered values of hemoglobin lesser than 10 mg/dL. High values of PLTs were detected only in 14 (10.68%) CML patients whereas the induced number of PLTs was observed in 3 (14.28%) CML patients.

**Table 1:** Physical and clinical features of CML patients.

Characteristics	Number (N = 21)	Percentage (%)
**Sex distribution**		
Male	11	52.38
Female	10	47.62
**Age-wise distributions**		
15–30	3	14.28
31–45	10	47.62
46–60	8	38.09
**Sign and symptoms**		
Anxiety	13	61.90
Depression	12	57.14
Swelling on body	13	61.90
Spleen enlargement	10	47.62
Liver enlargement	11	52.38
**WBC**		
<4–11 × 10^3^/µL	2	9.5
11.1–50 × 10^3^/µL	17	80.95
>50 × 10^3^/µL	2	9.52
**Hemoglobin**		
<10 mg/dL	13	61.90
>13 mg/dL	8	38.09
**Platelets**		
<150 × 10^3^/µL	2	9.52
150–400 × 10^3^/µL	16	76.19
>400 × 10^3^/µL	3	14.28

CML = chronic myelogenous leukemia, WBC = white blood cell.

Table 2 demonstrates that most of the 16 (76.19%) CML patients are in the chronic and 01 (4.76%) were in the blast Phase. However, 16 (76.19%) CML patients exhibit standard translocations and only one CML patient shows additional chromosomal abnormalities with Complex Variant Translocations. The hematological features of the patient was WBC 370 × 10^3^/µL, red blood cell 2.5 × 10^3^/µL, Hbg 6.6 g/dL, PLT 287 × 10^3^/µL, MCV 91/FL, MCH 26/pg, HCT (23%), MCHC 39.9 g/dL, neutrophils 52%, lymphocytes 01, eosinophils 03%, monocytes 05%, basophils 02%, promyelocytes 07%, myelocytes 15% meta-myelocytes 09% blast cells less then (5%). Cytogenetic bone marrow karyotyping result of the patient showed 46XX, t(1;9;22),) (q23;q34;q11.2) (Fig. S2, Supplemental Digital Content, http://links.lww.com/MD/H868). The patient was surviving in CP treated with Gleevec 400 mg/day.

**Table 2:** Cytogenetic and FISH results of CML patients.

Variables	Number	%
Standard translocations (9;22)	16	76.19
Complex variant translocations together with additional chromosomal abnormalities	01	4.76
Ph (–ve) (9;22) (q34; q11)	04	19.04
**The phase of CML dis**		
Chronic phase	16	76.19
Blast phase	01	4.76
**FISH analysis (N = 15**)		
FISH (+)	11	52.38
FISH (–)	4	19.04

CML = chronic myelogenous leukemia, FISH = fluorescence in situ hybridization.

**Figure 1.**
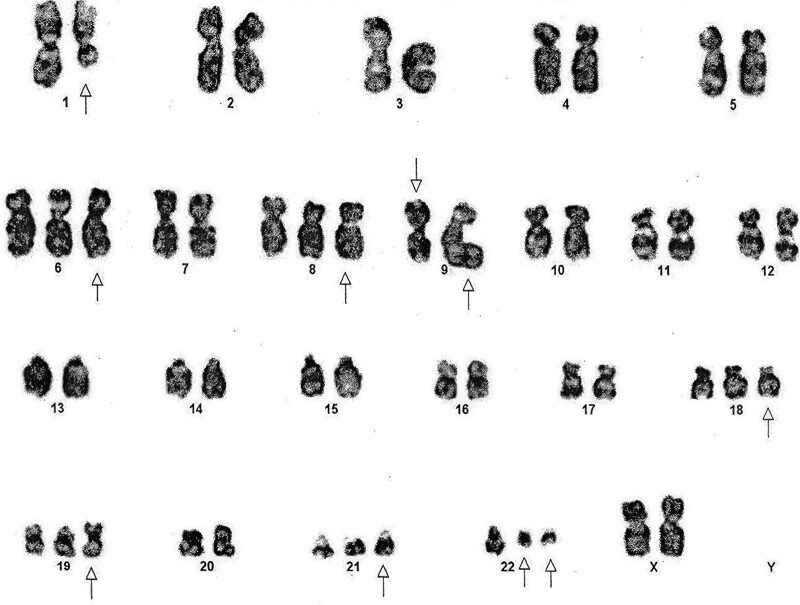
Cytogenetic analysis containing a variant three-way 06 additional chromosome 52XX, t (1;9;22) (q23.3; q34; q11.2), +6, +8, i(9) (q10;q10), +18,+19,+21 + der22 t(9;22)(q34;q11)[20] in blast phase.

Because of regular pain, fever, massive splenomegaly, and weight loss doctor recommends bone marrow aspiration and cytogenetic tests for follow-up. The results of peripheral blood counts of bone marrow show that WBC was 67 × 10^3^/µL, red blood cell 3.1 × 10^3^/µL, hemoglobin 7.4 g/dL, PLTs 25 × 10^3^/µL, MCV 109/FL, MCH 31.6/pg, HCT 25.5%, blast cells were 82% and the patient was migrating from first chronic to blast phase third phase of the disease. Cytogenetic karyotyping result of the patient showed 52XX, t (1;9;22) (q23.3; q34; q11.2), +6, +8, i(9) (q10;q10), +18,+19,+21 + der22 t(9;22)(q34;q11) (Fig. 1) (Fig. S1, Supplemental Digital Content, http://links.lww.com/MD/H867). All cells showed translocation among chromosomes 1q23, 9q34, and 22q11.2, +6, +8 an isochromosome 9, +18, +19, +21, and a derivative chromosome 22 generated by a translocation between chromosomes 9q34 and 22q11.2. Results of immunohistochemically stains on blast cells show TdT; diffuse positive, PAX-5; diffuse positive; CD34, positive, CD3; positive MPO; negative and CD117 also showed negative results. Before shifting to the second-generation drug Nilotinib (Tasigna) the patient died. After fluorescence in situ hybridisation analysis of 21, 11 CML patients shows BCR–ABL (+) gene fusion however, 04 showed BCR–ABL (–) and gene fusion.

## 4. Discussion

(9;22) (q34; q11) is established in thereabouts 90% of CML patients, on the other hand, complex/variant chromosome translocation is observed only in nearly 5% to 8% of cases. In such cases, additional chromosomes other than 9;22 are tangled.^[3,15,16]^ Extra genetic discrepancies happen in <10% of CML cases during diagnosis. The most common chromosomal aberrations in addition to a Ph-chromosome are + 19, +8, and + Ph,), However, –Y, +21, +17, −7, and − 17 were also found in fewer CML patients.^[6,17]^ Cytogenetic abnormalities like trisomy 8 (+8) and an extra copy of the Philadelphia chromosome are more common in CML cases.^[18]^

In CML, overall, 41 cases of 52 chromosomes with additional 06 chromosomes have been reported. However, 52 chromosomes with 06 additional chromosomes and the three-way translocation at the same patient in CML have been reported twice earlier in the available literature.^[19,20]^

In this we investigate a new case of 52, XX, t(1;9;22), +6 + 8,(q10;q10), +18 + 19, +21 + der22 t(9;22) (q34;q11) [20] in CML patient was cross checked at Mitel man Database of Chromosome Aberrations and Gene Fusions in Cancer.

Nearly 30% of CML patients in accelerated and 71% to 80% and CML patients in BP have additional chromosomal abnormalities. Among the various additional chromosomal abnormalities, trisomy 08 and an additional copy of the Ph chromosome are usual in CML cases. Diverse additional chromosomal abnormalities have been exposed to be linked with diverse effects on drug response and survival. More or less ACAs are linked with disease advancement and drug resistance, while they may additionally just reflect the hereditary unpredictability prompted by incessant activation of BCR–ABL1.^[18,21]^

Imatinib Mesylate (IM) is the first-line therapy for CML. IM is a BCR–ABL protein tyrosine kinase inhibitor against c-Abl, BCR/ABL. IM exposed significant effects in all phases of CML. The prescribed dose 400 mg/day for CP CML and 600 mg/day for the accelerated phase is recommended.^[13,22]^ In this study all the patients were treated with Imatinib Mesylate. Recently other protein tyrosine kinase inhibitors drugs approved by the FDA, and EMA like dasatinib, nilotinib, and bosutinib are used for the treatment of CML.^[23]^ Imatinib Mesylate is the first generation drug, however dasatinib, nilotinib and bosutinib are second generation tyrosine kinase inhibitors drugs.^[23]^

## Acknowledgments

We are thankful to the ORIC BUITEMS, Quetta, and Faculty of Life Sciences BUITEMS, Quetta for providing funding for this research. Under (Ph.D. Registration ID no. 27934).

## Supplementary Material


