# Supplementary material for: Cytogenetical and hematological analysis of chronic myelogenous leukemia patients with a novel case 52XX, t (1;9;22) (q23.3; q34; q11.2), +6, +8, i(9) (q10;q10), +18,+19,+21+der22 t(9;22)(q34;q11)
Source: Medicine (Baltimore). 2022 Nov 11;101(45):e31670. doi: 10.1097/MD.0000000000031670 (PMC9666132; doi:10.1097/MD.0000000000031670)
Supplement: Supplementary file 1 [file medi-101-e31670-s001.pdf]

Figure 1. **Supplementary**
46XX, t(9;22),) (q23;q34;q11.2)[20] 20 Cells were counted, all cells were positive for Philadephia chromosomes. (Case comments: Translocation between chromosome 9q34 and 22q11.2, resulting in Philadelphia chromosome.

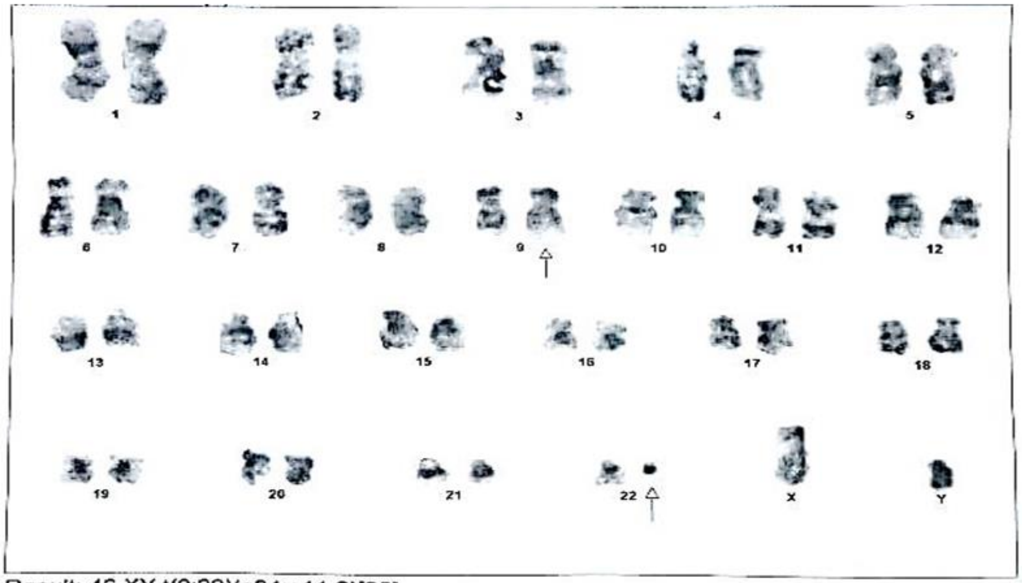

Result: 46,XY,t(9;22)(q34;q11.2)[20]
20 cells were counted, all cells were positive for Philadelphia chromosome.
Case comments: Translocation between chromosome 9q34 and 22q11.2, resulting in Philadelphia chromosome.
