# Supplementary material for: Cytogenetical and hematological analysis of chronic myelogenous leukemia patients with a novel case 52XX, t (1;9;22) (q23.3; q34; q11.2), +6, +8, i(9) (q10;q10), +18,+19,+21+der22 t(9;22)(q34;q11)
Source: Medicine (Baltimore). 2022 Nov 11;101(45):e31670. doi: 10.1097/MD.0000000000031670 (PMC9666132; doi:10.1097/MD.0000000000031670)
Supplement: Supplementary file 2 [file medi-101-e31670-s002.pdf]

Figure 2: **Supplementary**
52XX, t (1;9;22) (q23.3; q34; q11.2), +6, +8, i(9) (q10;q10), +18,+19,+21+der22 t(9;22)(q34;q11.2)[20] 20 ccll were counted, all cells were positive for double Philadelphia chromosome. Comment: All cells showed translocation among chromosomes 1q23, 9q34 and 22q11.2+6,+8, and iso chromosome 9,+18,+19,+21 and a derivative chromosome 22 generated by translocation between chromosomes 9q34 and 22q11.2

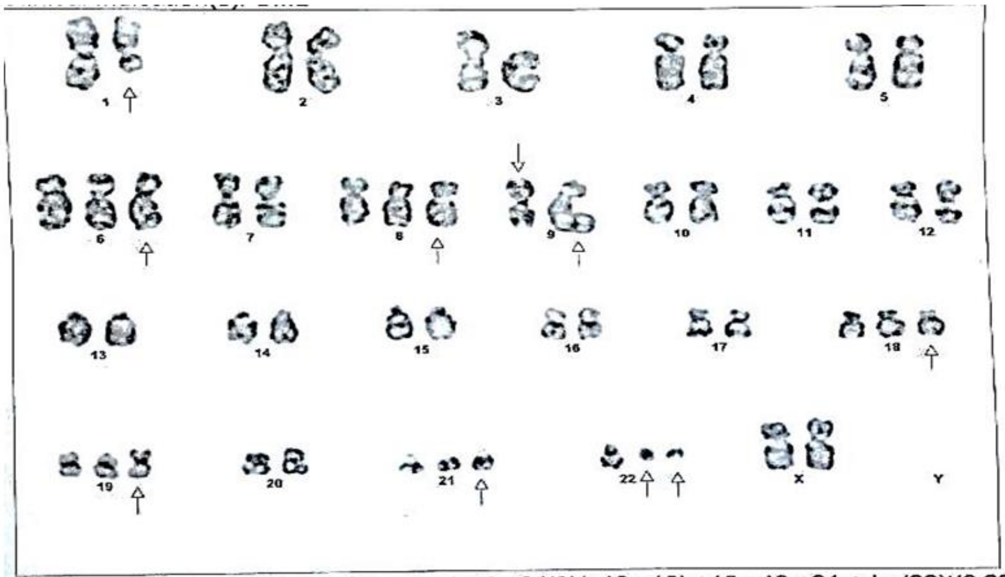

Result: 52,XX,t(1;9;22)(q23;q34;q11.2),+6,+8,i(9)(q10;q10),+18,+19,+21,+der(22)t(9;22)(q34;q11.2)[20]
20 cells were counted, all cells were positive for double Philadelphia chromosome.
Case Comment: All cells showed translocation among chromosomes 1q23, 9q34 and 22q11.2, +6,+8, an iso chromosome 9,+18,+19,+21 and a derivative chromosome 22 generated by translocation between chromosomes 9q34 and 22q11.2.
